# Nipah virus matrix protein promotes NF-κB activation by targeting multiple signaling modulators

**DOI:** 10.1128/jvi.00185-26

**Published:** 2026-05-21

**Authors:** Chang Ye, Xiaoyu Ma, Xianliang Ke, Zhongzi Yao, Renyi Liu, Tian Li, Peilu Zhang, Bayeta Senbeta Wakjira, Shixiang Tang, Feng Liu, Zi Ye, Ying Xie, Quanjiao Chen

**Affiliations:** 1 State Key Laboratory of Virology and Biosafety, Wuhan Institute of Virology, Chinese Academy of Sciences 74614 , Wuhan, China; 2 University of Chinese Academy of Sciences, Beijing, China; 3 Yangtze University 47897, Jingzhou, China; 4 Hubei University, Wuhan, China; University of Kentucky College of Medicine, Lexington, Kentucky, USA

**Keywords:** Nipah virus, matrix protein, NF-κB pathway, TRIM25, cellular signaling molecules

## Abstract

The Nipah virus (NiV) matrix (M) protein, essential for viral assembly, has been increasingly recognized for its potential immunomodulatory functions. However, its role in modulating the nuclear factor kappa-light-chain-enhancer of activated B cells (NF-κB) signaling pathway—a central regulator of inflammation and innate immunity—remains poorly defined. Here, we demonstrate that NiV-M significantly promotes activation of the classical NF-κB pathway. Our data showed that NiV-M interacts with multiple cellular signaling molecules of the NF-κB pathway, including tripartite motif containing 25 (TRIM25), inhibitor of nuclear factor kappa-B kinase subunit alpha (IKKα), nuclear factor of kappa light polypeptide gene enhancer in B-cell inhibitor alpha (IκBα), and the p65 subunit of NF-κB. Further analyses revealed that NiV-M enhanced the E3 ubiquitin ligase activity of TRIM25, facilitating its interaction with both retinoic acid-inducible gene I (RIG-I) and TNF receptor-associated factor 2 (TRAF2), thereby promoting K63-linked ubiquitination of RIG-I and TRAF2. Moreover, NiV-M strengthened the interaction between IKKα and IKKβ, leading to enhanced IKK complex activity and accelerated degradation of IκBα. In addition, NiV-M promoted phosphorylation and nuclear translocation of p65, thereby amplifying NF-κB-driven gene expression. In summary, our results demonstrate a multifaceted strategy by which NiV-M regulates the NF-κB pathway, a mechanism that may contribute to NiV pathogenesis via inflammatory dysregulation. These findings suggest potential therapeutic approaches for alleviating symptoms associated with immunopathology of NiV by targeting virus–host interactions.

**IMPORTANCE:**

This study reveals a previously unknown role of the Nipah virus matrix (M) protein in driving excessive inflammation, a key factor in the virus’s high mortality. We discovered that the M protein acts as a master switch, hijacking a central human immune pathway (NF-κB) at multiple points to trigger a “cytokine storm.” This explains how the virus causes severe tissue damage and organ failure in infected individuals. By identifying the specific human proteins targeted by the M protein, our work establishes that this viral component functions not only in viral assembly but also in the dysregulation of host inflammatory responses, thereby providing a new perspective on its potential contribution to severe NiV-associated disease. These findings open new avenues for treating NiV infections by developing drugs that target these interactions, potentially controlling the devastating inflammation rather than just the virus itself.

## INTRODUCTION

Nipah virus (NiV) is a highly pathogenic zoonotic virus belonging to the *Henipavirus* genus within the Paramyxoviridae family. NiV is a non-segment, single-stranded, negative-sense RNA virus. Since its initial identification during an outbreak in Malaysia in 1998–1999, recurrent outbreaks in Bangladesh, India, and other regions of Southeast Asia have been reported, with mortality rates ranging from 40% to 90% (1–3). Its genome encodes six structural proteins, namely, nucleoprotein (N), phosphoprotein (P), matrix protein (M), fusion protein (F), glycoprotein (G), and the large polymerase protein (L) and three non-structural proteins (V, W, and C, generated from the P gene through mRNA editing and alternative start codon) (4, 5). The hallmark pathological features of NiV infection include systemic vasculitis and severe inflammation with necrosis in the central nervous system (CNS) (6, 7). Hyperactivation of proinflammatory cytokines is a characteristic of NiV infection, leading to cytokine storm syndrome, which contributes to vascular leakage, multi-organ failure, and fatal encephalitis (8, 9). Clinical studies of outbreaks have reported elevated IL-6 and IL-10 levels in fatal cases, correlating with disease severity (10–13). Similarly, during SARS-CoV-2 infection, critically ill patients exhibited significantly elevated cytokine and chemokine levels, driving cytokine storms that result in multi-organ damage and high mortality. Notably, cytokine storms were also identified as a major contributor to the high fatality rates during the 1918 H1N1 influenza pandemic (14). This pathological phenomenon primarily arises from viral protein-mediated hyperactivation of the nuclear factor-κB (NF-κB) pathway. NF-κB is a major component that governs cytokine storm development. Notably, inhibition of NF-κB signaling can simultaneously suppress the release of multiple proinflammatory cytokines and chemokines, thereby reducing both morbidity and mortality in severe viral infections (15).

Among the aforementioned viral proteins, NiV-M is essential for viral assembly and budding (16, 17), and increasing evidence indicates that it may also modulate host immune responses (18). Nevertheless, the influence of NiV-encoded proteins on the NF-κB signaling pathway remains insufficiently characterized.

The NF-κB pathway comprises a family of evolutionarily conserved transcription factors that play central roles in immune regulation, inflammation, cell survival, and proliferation (19), as well as in host defense against pathogens, including viruses. NF-κB activation occurs through two distinct mechanisms: the classical and the alternative pathways (19). In the inactive state, the inhibitory IκB (IκBα) protein binds and sequesters NF-κB dimers in the cytoplasm (20). The classical pathway is typically activated by pro-inflammatory cytokines or pathogen-associated molecular patterns (PAMPs). Activation induces the IκB kinase (IKK) complex, composed of the catalytic subunits IKKα and IKKβ and the regulatory subunit IKKγ (also termed NEMO). This leads to phosphorylation of IκBα at serine residues Ser32/36, which targets it for ubiquitination and proteasomal degradation (21–23). Degradation of IκBα releases NF-κB dimers, predominantly RelA/p65 and p50, enabling their nuclear translocation and the subsequent transcription of genes involved in immune regulation, survival, and inflammation. Post-translational modifications of p65, including phosphorylation and acetylation, further modulate its transcriptional activity and interactions with coactivators (24–26). Formation of IKKα-IKKβ heterodimers is indispensable for classical pathway activation (20, 27). In contrast, the alternative pathway is activated by specific members of the TNF cytokine family and depends on IKKα homodimers for processing p100 to p52, thereby enabling formation and nuclear translocation of RelB/p52 complexes that regulate a distinct subset of genes (20, 27). Although traditionally considered independent, growing evidence indicates crosstalk and functional overlap between the two pathways, particularly involving IKKα (28).

Viruses have evolved sophisticated strategies to manipulate the NF-κB pathway, enabling them to evade host immune responses and establish conditions favorable for replication. In some cases, viral infection activates NF-κB to enhance cell survival and create a conducive environment for viral transcription and replication. In others, viruses inhibit NF-κB activation to suppress the host antiviral response. For instance, the Tat protein, essential for transcriptional activation of HIV-1, promotes NF-κB activation by directly interacting with TRAF6, thereby enhancing the oligomerization and auto-ubiquitination of TRAF6, and the synthesis of the K63-linked polyubiquitin chain, ultimately stimulating HIV-1 transcription (29). Similarly, the transcriptional activator Tax encoded by human T-cell leukemia virus type 1 (HTLV-1), which regulates viral gene expression and replication, interacts with several NF-κB pathway components, including p65, p50, and IκBα (30). Tax activates both axes of the NF-κB signaling pathway through the assembly of distinct Tax/IKK complexes. In the classical pathway, Tax stimulates activation of the IKK kinase complex, thereby triggering NF-κB signaling (31–34). In the alternative pathway, interaction between Tax and IKKγ facilitates the assembly of the Tax/IKKα complex, bypassing the requirement for NIK in IKK activation (35, 36). Constitutive NF-κB activation is a principal determinant of the oncogenic potential of Tax (37). These interactions underscore the pivotal role of the IKK complex in antiviral defense and highlight the diverse mechanisms through which viruses subvert host signaling. Understanding these processes provides critical insights into viral pathogenesis and may identify potential therapeutic targets.

Tripartite motif-containing protein 25 (TRIM25) is an E3 ubiquitin ligase that plays a crucial role in innate immune response against viral infections. TRIM25 catalyzes K63-linked polyubiquitination of retinoic acid-inducible gene I (RIG-I) (38, 39), a cytosolic pattern recognition receptor that detects viral RNA, leading to the activation of downstream signaling cascades, including NF-κB and interferon regulatory factor 3 (IRF3), and the production of type I interferons (38), which is one of the defining features of TRIM25. In addition to its role in RIG-I signaling, TRIM25 regulates NF-κB activation via interaction with TRAF2. By enhancing K63-linked polyubiquitination of TRAF2, TRIM25 promotes recruitment and activation of downstream kinases such as TAK1 and IKKβ, thereby facilitating NF-κB signaling (40). Viruses have developed mechanisms to counteract TRIM25-mediated antiviral responses. For example, the NS1 protein of influenza A virus binds to the coiled-coil domain of TRIM25, preventing its dimerization and subsequent activation of RIG-I signaling (41). Similarly, the nucleocapsid protein of SARS-CoV interacts with the SPRY domain of TRIM25, inhibiting its ability to activate RIG-I (42). These interactions highlight the strategic targeting of TRIM25 by viruses to evade host immune responses.

Given the central role of NF-κB in inflammation, antiviral immunity, and cell survival, elucidating how NiV proteins manipulate this pathway is essential for understanding NiV pathogenesis and developing therapeutic interventions. Despite extensive studies on viral modulation of NF-κB, the mechanisms by which NiV engages this pathway remain unclear. Specifically, it is unknown whether NiV targets upstream sensors (e.g., RIG-I/MDA5), adaptor proteins, or the IKK complex. Paradoxically, although NF-κB activation typically restricts viral replication, numerous viruses exploit this pathway to establish a proviral environment, suggesting a delicate balance between host defense and viral adaptation. To address these gaps, in this study, we aimed to investigate the functional relationship between NiV infection and NF-κB activation, with particular emphasis on the roles of individual viral proteins in modulating this critical immune regulatory pathway. A particular emphasis was placed on the TNFα- and virus-induced canonical pathway. Furthermore, we investigated the mechanisms by which NiV-M influences NF-κB signaling. The rationale was to obtain mechanistic insights into viral modulation of host immune responses, which may inform the development of novel therapeutic interventions.

## RESULTS

### M significantly promotes activation of the NF-κB pathway

**Fig 1:**
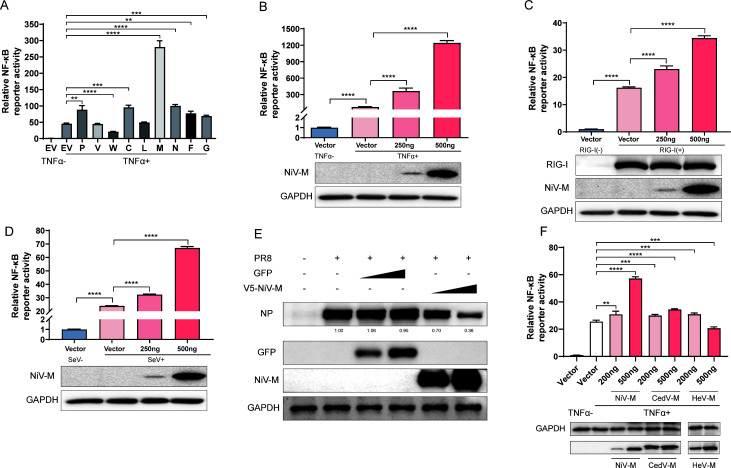
NiV-M affects the TNFα- and virus-induced activation of the NF-κB pathway. (**A**) HEK293T cells were transfected with an empty vector (EV) or plasmids encoding the indicated NiV proteins, together with the NF-κB-firefly luciferase reporter and the internal control Renilla luciferase plasmid pRL-TK. At 24 h post-transfection (hpt), cells were mock-stimulated or stimulated with TNFα (10 ng/mL) for 8 h before luciferase reporter assays were performed. (**B–D**) Cells were transfected with the NF-κB luciferase reporter and the internal control Renilla luciferase, as well as an empty vector or an increasing amount of NiV-M plasmid, and then either mock-stimulated or stimulated with TNFα (10 ng/mL) (**B**), RIG-I (**C**), or SeV (100 HA units/mL) (**D**). (**E**) A549 cells were transfected with increasing amounts of NiV-M or GFP control plasmid for 20 h and then either mock-infected or infected with PR8 (MOI = 0.01) for 12 h. Cells were collected for Western blotting. NP levels were normalized to GAPDH, and values are presented as fold change relative to the mock group (set to 1). (**F**) The experiment was performed as described in B, except with increasing amounts of Flag-NiV-M, Flag-CedV-M, and Myc-HeV-M. Data are presented as the fold induction of NF-κB activity relative to the mock-stimulated EV control (set to 1) from three independent experiments (mean ± SD). **P < 0.01, ***P < 0.001, ****P < 0.0001 (Student’s t-test).

As the NF-κB pathway is one of the most classical inflammatory signaling cascades, we investigated whether NiV proteins contribute to NF-κB-mediated inflammatory responses induced by TNFα and RIG-I. All NiV-encoded proteins were screened using dual-luciferase reporter assays. HEK293T cells were co-transfected with NF-κB-Luc, the internal control Renilla luciferase, and plasmids encoding the candidate viral proteins, followed by stimulation with TNFα. As shown in Fig. 1A, P, C, M, N, F, and G proteins significantly enhanced TNFα-induced activation of the NF-κB promoter, whereas W suppressed inflammatory responses. Because M displayed the most pronounced fold-change in reporter activity, it was selected as the focus to investigate the relationship between NiV and NF-κB signaling. In parallel, we examined the effect of M on RIG-I- and Sendai virus-mediated NF-κB activation and NF-κB-dependent proinflammatory cytokine expression. The results demonstrated that NiV-M could promote TNFα-, RIG-I-, and virus-induced NF-κB activation in a dose-dependent manner (Fig. 1B and D). Consistently, RNA levels of NF-κB-dependent proinflammatory cytokines increased proportionally with NiV-M expression (Fig. S1). To validate the biological impact of NiV-M overexpression on viral infectivity, we transfected A549 cells with increasing doses of NiV-M-encoding plasmid or GFP control plasmid, followed by infection with influenza A virus (PR8). Western blot analysis of the viral nucleoprotein (NP) showed that NiV-M expression significantly reduced PR8 NP protein accumulation in a dose-dependent manner compared with the empty vector control, whereas GFP expression exerted no discernible impact (Fig. 1E). This result indicates that the cellular environment shaped by NiV-M overexpression restricts, rather than promotes, subsequent viral infection in this experimental system.

### NiV-M is the most potent activator of NF-κB among henipavirus M proteins

To investigate whether NF-κB activation capacity is conserved among paramyxovirus M proteins, we compared M proteins from the highly pathogenic Nipah virus (NiV) and Hendra virus (HeV), as well as the low-pathogenicity Cedar virus (CedV). In dual-luciferase reporter assays, at low transfection doses, all three M proteins induced similar, modest NF-κB activation (≈1.2-fold enhancement) (Fig. 1F). In contrast, at high transfection doses, striking differences in activation capacity emerged: NiV-M mediated the strongest activation (≈2.0-fold enhancement), followed by CedV-M (≈1.36-fold enhancement), whereas HeV-M activity was suppressed, falling slightly below baseline levels (≈0.8-fold, a 1.25-fold reduction).

This functional divergence likely reflects an evolutionary trade-off shaped by viral adaptation to distinct host species (e.g., fruit bats, horses, pigs, humans). Efficient human-to-human transmission of NiV may necessitate robust immunomodulatory capacity to establish productive infection and drive severe inflammation, a process that can facilitate viral spread. By contrast, HeV has a distinct transmission chain and ecological niche, and HeV-M may have evolved to prioritize alternative functions such as immune evasion. The weak NF-κB activation of CedV-M is consistent with the viral lack of severe pathogenicity in its natural host.

### M facilitates NF-κB signaling pathway at the level of the p50/p65

**Fig 2:**
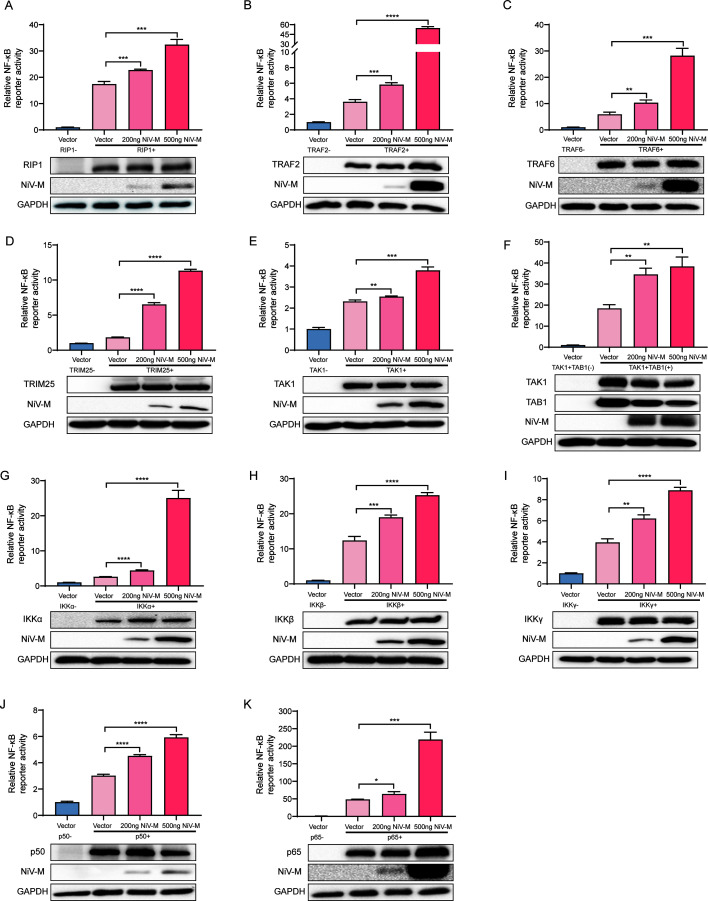
Identification of the cellular signaling molecules targeted by NiV-M in the NF-κB pathway. HEK293T cells were transfected with NF-κB-Luc (50 ng) and RL-TK (10 ng) together with Myc-RIP1 (200 ng) (**A**), Flag-TRAF2 (200 ng) (**B**), HA-TRAF6 (200 ng) (**C**), Flag-TRIM25 (200 ng) (**D**), Flag-TAK1 (200 ng) (**E**), Flag-TAK1/HA-TAB1 (each 100 ng) (**F**), HA-IKKα (200 ng) (**G**), Flag-IKKβ (200 ng) (**H**), HA-IKKγ (200 ng) (**I**), Flag-p50 (200 ng) (**J**), and HA-p65 (200 ng) (**K**), together with V5-NiV-M or empty vector (corresponding amounts). At 24 hpt, cells were harvested, and luciferase activity was measured. Expression of signaling molecules and NiV-M was analyzed by Western blotting using anti-Myc, anti-Flag, anti-HA, or anti-V5 antibodies. Data are presented as mean ± SD from three independent experiments. *P < 0.05, **P < 0.01, ***P < 0.001, ****P < 0.0001 (Student’s t-test).

To elucidate the molecular mechanism by which NiV-M activates the TNFα/RIG-I-induced NF-κB signaling pathway, we evaluated the functional contributions of key cellular signaling molecules in the NF-κB pathway to NiV-M-driven NF-κB activation. NF-κB-Luc activities induced by 11 factors (RIP1, TRAF2, TRAF6, TRIM25, TAK1, TAB1, IKKα, IKKβ, IKKγ, p50, and p65) representing distinct steps of the signaling cascade were examined. Dual-luciferase reporter assays revealed that NF-κB activation elicited by NiV-M was promoted at every step (Fig. 2), indicating that NiV-M facilitates NF-κB activation at the level of p50/p65.

### M interacts with TRIM25, IKKα, IκBα, and p65

**Fig 3:**
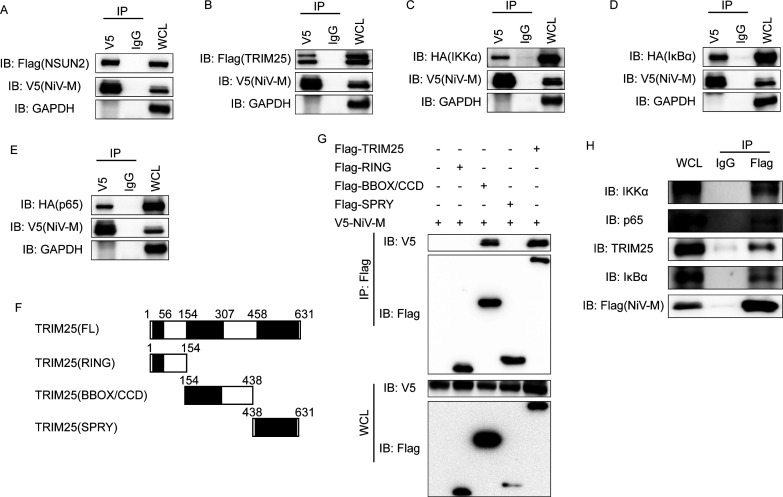
M interacts with TRIM25, IKKα, IκBα, and p65. HEK293T cells were co-transfected with V5-NiV-M and Flag-NSUN2 (**A**), Flag-TRIM25 (**B**), HA-IKKα (**C**), HA-IκBα (**D**), or HA-p65 (**E**) expression plasmids. At 24 hpt, cells were lysed, and lysates were immunoprecipitated using anti-V5 antibody-conjugated beads (IP: V5) or non-specific IgG (IP: IgG) as a control. (**F**) Schematic illustration of TRIM25 and its truncated mutants. (**G**) HEK293T cells were co-transfected with V5-NiV-M and indicated plasmids. At 24 hpt, cell lysates were immunoprecipitated using anti-Flag antibody-conjugated beads. (**H**) HEK293T cells were transfected with Flag-NiV-M and harvested at 24 hpt. Lysates were immunoprecipitated using anti-Flag antibody-conjugated beads. For all panels, whole-cell lysates (WCL) and IP complexes were analyzed by Western blotting using the indicated antibodies. IgG, immunoglobulin G control.

Since the results indicated that NiV-M promotes NF-κB activation at multiple steps, we further investigated interactions between NiV-M and 13 cellular signaling molecules (the 11 factors described above plus RIG-I and IκBα) using co-IP assays. Cellular signaling molecules tagged with Myc, HA, or Flag were each co-transfected with a plasmid expressing V5-NiV-M into HEK293T cells. To validate the specificity of our co-IP system, we initially confirmed the interactions between NiV-M and its well-characterized interacting partners NSUN2 and RAD18, which were employed as positive controls (Fig. 3A and Fig. S2A) (43, 44). Under uniform experimental parameters, exogenously NiV-M was found to interact with TRIM25, IKKα, IκBα, and p65 (Fig. 3B and E), whereas no binding was observed between NiV-M and RIP1, TRAF2, RIG-I, TRAF6, TAK1, TAB1, IKKβ, IKKγ, or p50 (Fig. S2B through J).

To verify the NiV-M-TRIM25 interaction, plasmids expressing HA- or V5-tagged NiV-M were co-transfected with a plasmid expressing Flag-TRIM25 in HEK293T cells. Co-IP was then performed with anti-HA (IP: HA) or anti-Flag (IP: Flag) antibody-conjugated beads, showing the association of NiV-M with Flag-TRIM25 (Fig. S2, K and L). To map the TRIM25 domain required for this interaction, three TRIM25 truncation mutants were generated (Fig. 3F). The results revealed that M could interact with the BBOX/CCD domain of TRIM25 (Fig. 3G). Interestingly, while the BBOX/CCD domain of TRIM25 was co-precipitated with NiV-M, the amount of NiV-M pulled down by the BBOX/CCD domain was consistently lower than that precipitated by full-length TRIM25 in parallel experiments. This observation suggests that the BBOX/CCD domain, while necessary for the NiV-M/TRIM25 interaction, may not be the sole determinant. Additional regions within TRIM25 likely provide secondary contact sites that stabilize the NiV-M/TRIM25 complex, thereby enabling more efficient co-precipitation of NiV-M by full-length TRIM25. Similarly, reverse co-IP experiments confirmed interactions between Flag-NiV-M and HA-tagged IKKα, IκBα, and p65 (Fig. S2M through O). To assess whether these interactions occur with endogenously expressed proteins under native conditions. The endogenous IP experiments were performed, and the results showed that NiV-M could interact with endogenous TRIM25, IKKα, IκBα, and p65 (Fig. 3H), consistent with the exogenous co-IP results. Collectively, these data indicate that NiV-M can engage multiple nodes of the NF-κB signaling pathway.

### M enhances the interaction between TRIM25 and RIG-I and promotes the ubiquitination of RIG-I

To explore the biological functions of the interaction between NiV-M and TRIM25, IKKα, IκBα, and p65, respectively. First, we tested the functions of the interaction between NiV-M and TRIM25. It is well established in the field that, upon viral infection, the E3 ubiquitin ligase TRIM25 could bind with RIG-I and mediate its K63-linked polyubiquitination, thereby facilitating NF-κB pathway activation (39). As mentioned above, because TRIM25 could interact with NiV-M, we examined whether NiV-M alters TRIM25-RIG-I interaction, thereby modulating NF-κB signaling.

**Fig 4:**
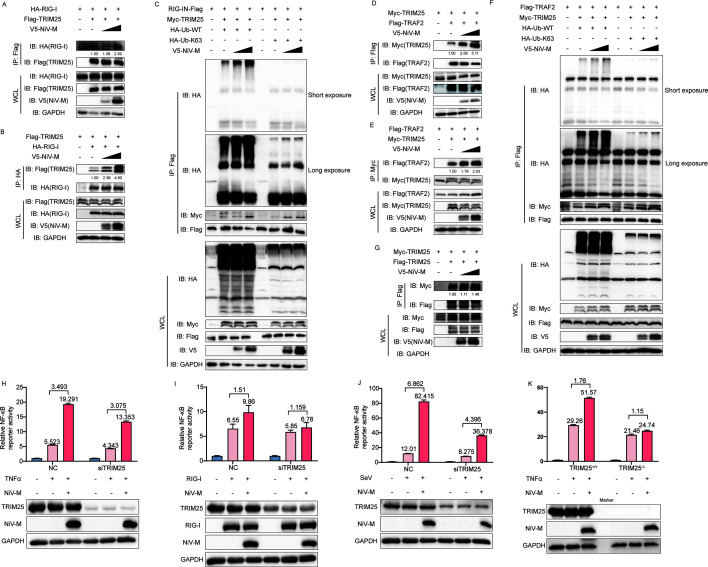
Effect of the NiV-M on TRIM25 function. HEK293T cells were co-transfected with Flag-TRIM25, HA-RIG-I, and V5-NiV-M or an empty vector for 24 h. Cell lysates were immunoprecipitated with anti-HA (IP: HA) (**A**) or anti-Flag (IP: Flag) antibody-conjugated beads (**B**). The intensity of co-precipitated RIG-I (**A**) and TRIM25 (**B**) was normalized to the immunoprecipitated TRIM25 (**A**) and RIG-I (**B**) in each lane. (**C**) Cells were co-transfected with RIG-IN-Flag, Myc-TRIM25 expression plasmid, and ubiquitin expression plasmid of HA-Ub-WT, HA-Ub-K63, along with V5-NiV-M expression plasmid or empty vector. At 24 hpt, cells were collected for immunoprecipitation with anti-Flag antibody-conjugated beads (IP: Flag). The expression of ubiquitinated proteins linked with RIG-IN was detected by anti-HA mAb. HEK293T cells were co-transfected with Myc-TRIM25, Flag-TRAF2, and V5-NiV-M or an empty vector for 24 h. Lysates were immunoprecipitated with anti-Flag (IP: Flag) (**D**) or anti-Myc (IP: Myc) antibody-conjugated beads (**E**). The intensity of co-precipitated TRIM25 (**D**) and TRAF2 (**E**) was normalized to the immunoprecipitated TRAF2 (**D**) and TRIM25 (**E**) in each lane. (**F**) HEK293T cells were co-transfected with Flag-TRAF2, Myc-TRIM25 expression plasmid, and ubiquitin expression plasmid of HA-Ub-WT, HA-Ub-K63, along with V5-NiV-M expression plasmid or empty vector. At 24 hpt, cells were collected for immunoprecipitation with anti-Flag antibody-conjugated beads (IP: Flag). The expression of ubiquitinated proteins linked with TRAF2 was detected using anti-HA mAb. (**G**) HEK293T cells were co-transfected with Myc-TRIM25, Flag-TRIM25, and increasing amounts of NiV-M. Co-IP was performed using anti-Flag antibody-conjugated beads. The intensity of co-precipitated Myc-TRIM25 was normalized to the immunoprecipitated Flag-TRIM25 in each lane. Cells were transfected with si-TRIM25 or siNC for 36 h, followed by transfection with a dual-luciferase reporter plasmid and a Flag-NiV-M expression vector. At 60 hpt, cells were stimulated with TNFα (10 ng/mL) (**H**), RIG-I (**I**), or SeV (100 HA unit/mL) (**J**) or mock-stimulated before luciferase reporter assays were performed. (**K**) WT and TRIM25-knockout (TRIM25-KO) cells were co-transfected as described in H. The values above individual bars represent the normalized ratio of dual-luciferase activity (Firefly/Renilla) for the NF-κB reporter assay, calculated relative to the untreated control group (set to 1). The levels of the individual proteins were detected by Western blotting. NC, negative control.

HEK293T cells were co-transfected with plasmids encoding HA-RIG-I, Flag-TRIM25, and either an empty vector or increasing amounts of a plasmid expressing V5-NiV-M. Protein interactions were assessed by co-IP using anti-Flag antibody-conjugated beads (TRIM25 pull-down) and reverse co-IP using anti-HA antibody-conjugated beads (RIG-I pull-down). The results showed that NiV-M could increase the interaction between TRIM25 and RIG-I (Fig. 4A and B). Since TRIM25-mediated K63-linked ubiquitination of RIG-I is essential for initiating host antiviral innate immunity through the RIG-I signaling pathway, we investigated whether NiV-M modulates the activation of RIG-I. HEK293T cells were co-transfected with RIG-IN-Flag, Myc-TRIM25, HA-tagged wild type (WT) or K63-linked ubiquitin plasmids, and empty vector or increasing amounts of V5-NiV-M. Whole-cell lysates were subjected to co-IP using anti-Flag antibody-conjugated beads, followed by immunoblot analysis. WT and K63-linked polyubiquitination of RIG-I were observed, and the levels increased along with the rising expression of NiV-M (Fig. 4C), suggesting that NiV-M promotes the TRIM25-mediated WT and K63-linked polyubiquitination of RIG-I.

### M enhances the interaction between TRIM25 and TRAF2 and promotes the ubiquitination of TRAF2

In the TNFα-induced NF-κB signaling pathway, TRIM25 interacts with TRAF2 and promotes the attachment of K63-linked polyubiquitin chains to TRAF2, thereby enhancing TNFα-induced NF-κB signal transduction. Based on this, we investigated the effect of NiV-M on the TRIM25-TRAF2 complex. To determine whether NiV-M modulates TRIM25-TRAF2 complex formation, HEK293T cells were co-transfected with plasmids encoding Myc-TRIM25 and Flag-TRAF2, together with either empty vector or increasing amounts of a plasmid encoding V5-NiV-M. Protein interactions were assessed by immunoprecipitation with anti-Flag antibody–conjugated beads (TRAF2 pull-down) and by reciprocal IP with anti-Myc antibody–conjugated beads (TRIM25 pull-down). The results demonstrated that NiV-M promotes the interaction between TRIM25 and TRAF2 (Fig. 4D and E).

Given that TRIM25 enhances K63-linked ubiquitination of TRAF2 to promote NF-κB activation (40), we next examined NiV-M’s role in this process. HEK293T cells were co-transfected with plasmids encoding HA-Ub-WT or HA-Ub-K63, together with plasmids encoding Myc-TRIM25, Flag-TRAF2, and empty vector or increasing amounts of plasmid-encoding V5-NiV-M. Ubiquitination of TRAF2 was analyzed by Flag immunoprecipitation followed by immunoblotting with anti-HA antibody. WT and K63-linked polyubiquitination of TRAF2 were observed, and the levels increased along with the rising expression of NiV-M (Fig. 4F), indicating that NiV-M promotes TRIM25-mediated WT and K63-linked polyubiquitination of TRAF2. Given that NiV-M interacts with the BBOX/CCD domain of TRIM25 (Fig. 3F), a region critical for mediating TRIM25 oligomerization (41), we investigated whether NiV-M influences TRIM25 oligomerization. HEK293T cells were co-transfected with Myc-TRIM25, Flag-TRIM25, and an increasing amount of V5-NiV-M. Co-IP using anti-Flag antibody–conjugated beads revealed enhanced pull-down of Myc-TRIM25 in the presence of NiV-M, indicating that NiV-M promotes TRIM25 oligomerization (Fig. 4G). Collectively, NiV-M promotes the formation of TRIM25-RIG-I and TRIM25-TRAF2 complexes by enhancing TRIM25 oligomerization, thereby augmenting K63-linked ubiquitination of RIG-I and TRAF2, thus promoting NF-κB pathway activation.

### TRIM25 is a key but not exclusive target of M in promoting NF-κB activation

To determine whether TRIM25 is the principal target through which NiV-M influences NF-κB activation, TRIM25 knockdown (KD) cells were generated. HEK293T cells were transfected with TRIM25-targeting siRNA or non-targeting control siRNA. After 36 hpt, cells were transfected with a dual-luciferase reporter plasmid together with a NiV-M expression vector. Following the indicated treatments, luciferase activity was measured to assess pathway activation. Dual-luciferase assays showed that NiV-M promoted NF-κB activation induced by RIG-I, SeV, and TNFα in both WT (consistent with Fig. 1B through D) and TRIM25 KD cells (Fig. 4H through J). Notably, although the NF-κB pathway remained activatable in TRIM25 KD cells, the magnitude of NiV-M-mediated activation was reduced relative to WT controls (Fig. 4H through J). Furthermore, we generated a TRIM25-knockout cell line and performed parallel NF-κB reporter gene assays. The results were consistent with the trends observed following TRIM25 knockdown: NiV-M still enhanced TNFα-induced NF-κB pathway activation in TRIM25-knockout cells (Fig. 4K). Together with the co-IP results in Fig. 3, these data indicate that NiV-M targets additional cellular signaling molecules (such as IKKα, IκBα, and p65) in both virus- and TNFα-induced NF-κB signaling. In summary, TRIM25 is a key, but not the exclusive, target through which NiV-M promotes NF-κB activation.

### M enhances the interaction between IKKα and IKKβ and facilitates IKK-dependent IκBα degradation

**Fig 5:**
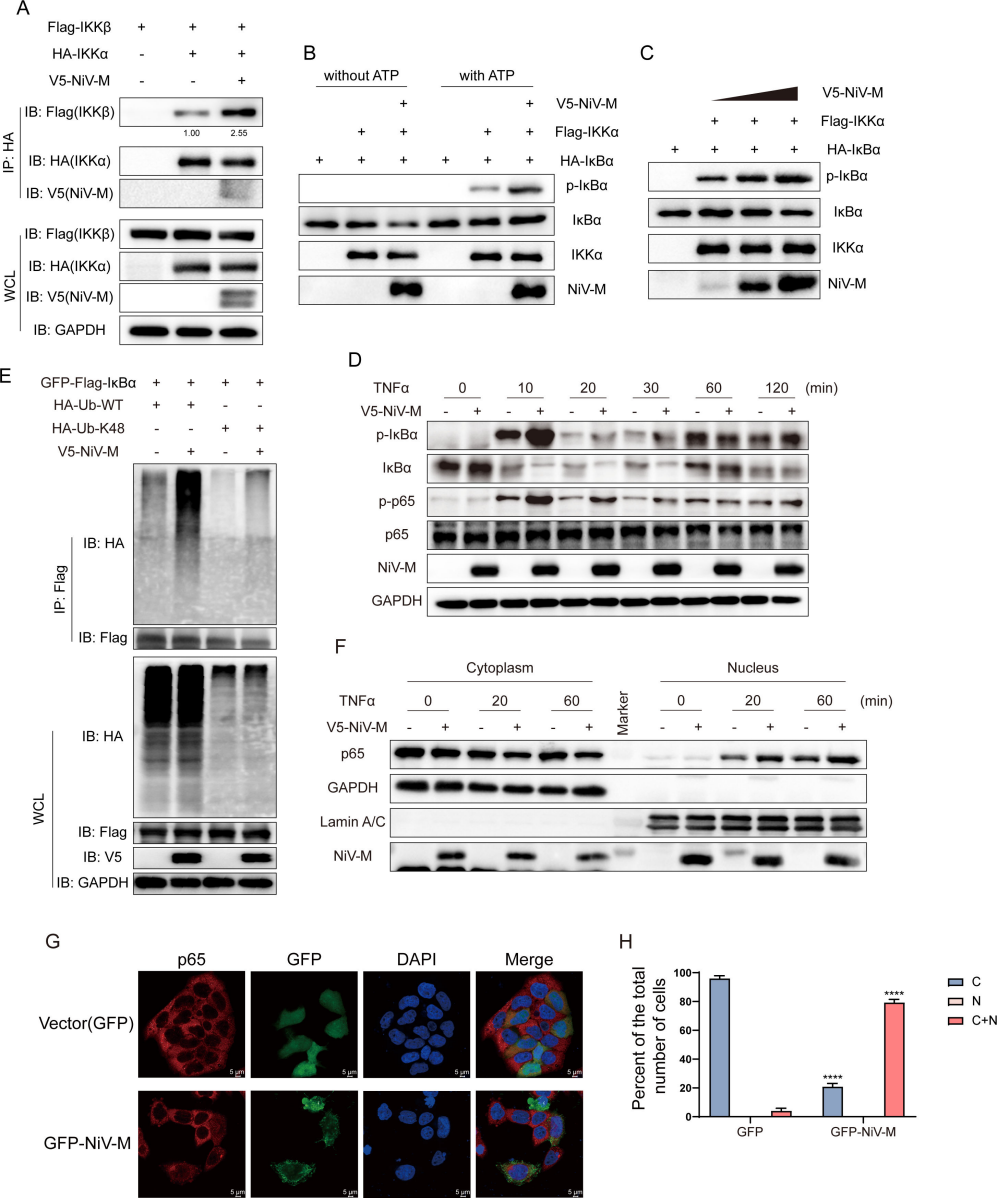
The effects of the interaction between NiV-M and IKKα, IκBα, or p65 on their function. (**A**) HEK293T cells were transfected with Flag-IKKβ, together with HA-IKKα and V5-NiV-M or an empty vector. Lysates were collected at 24 hpt and subjected to immunoprecipitation using anti-HA (IP: HA) antibody-conjugated beads. The intensity of co-precipitated IKKβ was normalized to the immunoprecipitated IKKα in each lane. (**B**) Cells were transfected with HA-IκBα, Flag-IKKα, or V5-NiV-M. At 36 hpt, proteins were immunoprecipitated with anti-Flag, anti-HA, or anti-V5 antibody. A kinase assay of the immunoprecipitated proteins *in vitro* was carried out with or without ATP and analyzed by immunoblotting. (**C**) The experiment was performed as described in B, except with increasing amounts of NiV-M. (**D**) HEK293T cells were transfected with an empty vector or a V5-NiV-M expression plasmid. At 24 hpt, cells were stimulated with 10 ng/mL TNFα for different times (0, 10, 20, 30, 60, and 120 min). Cells were lysed, and phosphorylation of IκBα and p65 was detected using rabbit anti-IκBα Ser32 pAb and rabbit anti-p65 Ser536 pAb, respectively. (**E**) Cells were co-transfected with GFP-Flag-IκBα expression plasmid and ubiquitin expression plasmids HA-Ub-WT or HA-Ub-K48, together with V5-NiV-M expression plasmid or empty vector. At 24 hpt, cells were collected for immunoprecipitation with anti-Flag antibody-conjugated beads (IP: Flag). (**F**) HEK293T cells were transfected with the empty vector or the V5-NiV-M plasmid. At 24 hpt, cells were stimulated with 10 ng/mL TNFα for 0, 20, or 60 min. Nuclear and cytoplasmic fractions were separated and analyzed by Western blotting using antibodies against p65, Lamin A/C (nuclear control), or GAPDH (cytoplasmic control). (**G**) HeLa cells were transfected with GFP-NiV-M or GFP empty vector. At 24 hpt, cells were immunostained for GFP (green) and p65 (red), with DAPI (blue) used to stain nuclear DNA. Imaging was performed with a scale bar set at 5 μm. (**H**) Percentage of cells with p65 expression in the cytoplasm (C), nucleus (N), or both compartments (C + N) in WT and NiV-M-transfected cells. Values represent the ratio relative to total counted cells (mean ± SD, *n* = 3 independent experiments, duplicate technical replicates). ****P < 0.0001.

After examining the role of NiV-M in modulating TRIM25 function, we next investigated its effects on IKKα, IκBα, and p65. Because classical NF-κB pathway activation involves IKKα interactions with TAK1, IKKβ, IKKγ, IκBα, and p65, we assessed whether NiV-M modulates these protein–protein interactions. HEK293T cells were co-transfected with plasmids encoding HA-TAK1 (Fig. S3A), Flag-IKKβ (Fig. 5A), HA-IKKγ (Fig. S3B), HA-IκBα (Fig. S3C), or HA-p65 (Fig. S3D), together with plasmids encoding Flag- or HA-tagged IKKα and V5-NiV-M. Co-IP assays were performed using anti-Flag or anti-HA antibody-conjugated beads. The results revealed that NiV-M significantly enhanced the interaction between IKKα and IKKβ (Fig. 5A).

Since phosphorylation of IκBα by the IKK complex is a critical step in NF-κB pathway activation (45), we hypothesized that NiV-M may directly target the IKK complex to enhance its kinase activity toward IκBα. To test this, we conducted an *in vitro* kinase assay using immunoprecipitated proteins (NiV-M, IκBα, and IKKα). The reactions were performed in the presence or absence of ATP to supply phosphate groups for the phosphorylation reaction, allowing detection of newly formed phosphorylated IκBα (p-IκBα). The results demonstrated that in the absence of ATP, only minimal levels of p-IκBα (Ser32) were detected. In contrast, the presence of ATP led to an increased level of p-IκBα when IKKα was added to the reaction. Notably, the NiV-M enhanced the phosphorylation of IκBα (Fig. 5B), indicating that NiV-M potentiates the kinase activity of IKKα toward its substrate IκBα. Moreover, increasing amounts of NiV-M resulted in a dose-dependent elevation of IKKα-induced p-IκBα levels (Fig. 5C).

As IKK activation typically triggers IκBα phosphorylation and subsequent degradation, we next examined the impact of NiV-M on IκBα stability. HEK293T cells were transfected with a plasmid encoding NiV-M or an empty vector and stimulated with TNFα for the indicated times. The results showed that at 10 min post-TNFα treatment, cells overexpressing NiV-M exhibited significantly higher p-IκBα levels compared to those of empty vector controls. Furthermore, total IκBα levels were markedly lower in NiV-M-expressing cells at 20 and 30 min after TNFα stimulation than those of empty vector (Fig. 5D). These findings indicate that NiV-M overexpression enhances TNFα-induced phosphorylation of IκBα, leading to a more rapid and pronounced degradation of IκBα and thereby promoting NF-κB release from its inhibitory complex.

It is well known that activated IKK complexes phosphorylate IκBα at serine residues 32 and 36, promoting its K48-linked polyubiquitination and subsequent degradation via the proteasome pathway (46). To assess whether NiV-M enhances IκBα polyubiquitination, we co-transfected HEK293T cells with plasmids encoding GFP-Flag-IκBα, together with either V5-NiV-M or empty vector, and plasmids encoding HA-Ub-WT or HA-Ub-K48. Co-IP was performed using anti-Flag antibody-conjugated beads. Both WT and K48-linked polyubiquitination of IκBα were observed, and the levels increased with NiV-M expression (Fig. 5E). These findings suggest that NiV-M enhances NF-κB activity by accelerating proteasome-mediated degradation of IκBα. Collectively, these results demonstrate that NiV-M facilitates IKK-dependent degradation of IκBα.

### M promotes p65 phosphorylation and nuclear translocation but does not influence the formation of the p65/p50 heterodimer

After exploring the role of NiV-M in regulating the function of TRIM25, IKKα, and IκBα, we next examined p65. Phosphorylation (e.g., at Ser536) and nuclear translocation of p65 are hallmarks of NF-κB activation. Upon TNFα stimulation, the IKKα/β complex phosphorylates IκBα, leading to the degradation of IκBα via K48 ubiquitination, thus releasing p65, enabling its nuclear entry and transcriptional activity. We therefore analyzed the impact of NiV-M on p65 phosphorylation and subcellular localization. As noted above, NiV-M could interact with p65. To investigate whether this interaction affects the binding of p65 and p50, HEK293T cells were co-transfected with an empty vector or a V5-NiV-M expression plasmid together with plasmids encoding Flag-p50 and HA-p65, and co-IP was performed with anti-Flag or anti-HA antibody-conjugated beads. The results showed that the amounts of p50 or p65 were not altered in the absence of NiV-M compared to that of empty vector (Fig. S3E and F), indicating that NiV-M does not affect p65/p50 heterodimer formation. We next examined whether the interaction between NiV-M and p65 influences p65 phosphorylation and nuclear translocation. HEK293T cells were transfected with NiV-M expression plasmid or empty vector, followed by TNFα stimulation for the indicated times. Total and phosphorylated p65 levels were then analyzed in whole-cell lysates, and nuclear and cytoplasmic fractionation experiments were performed to assess p65 subcellular localization. Phosphorylation of p65 was significantly increased in NiV-M-expressing cells at 10 and 20 min after TNFα stimulation compared to that of empty vector (Fig. 5D), indicating that NiV-M enhances TNFα-induced p65 phosphorylation. Nuclear-cytoplasmic fractionation further confirmed that NiV-M-expressing cells exhibited significantly increased p65 nuclear translocation relative to empty vector (Fig. 5F). In addition, confocal microscopy analysis revealed that p65 was predominantly localized to the cytoplasm in mock-transfected cells. In contrast, p65 exhibited a diffuse subcellular distribution in NiV-M-expressing cells, further confirming that NiV-M drives the nuclear translocation of p65, whereas GFP expression had no effect (Fig. 5G). To further confirm the role of NiV-M in promoting p65 nuclear translocation, we performed quantitative immunofluorescence analysis of p65 subcellular localization. In the GFP control group, p65 was almost exclusively cytoplasmic (approximately 96% of cells). In contrast, p65 was diffusely distributed in both the cytoplasm and nucleus of NiV-M-expressing cells. The percentage of cells displaying nuclear or nucleocytoplasmic p65 localization increased from roughly 4% in control cells to approximately 79% in cells expressing GFP-NiV-M. These quantitative data further demonstrate that NiV-M effectively promotes p65 nuclear translocation. Collectively, these results demonstrated that NiV-M promotes p65 phosphorylation and nuclear accumulation, thereby amplifying NF-κB signaling.

## DISCUSSION

**Fig 6:**
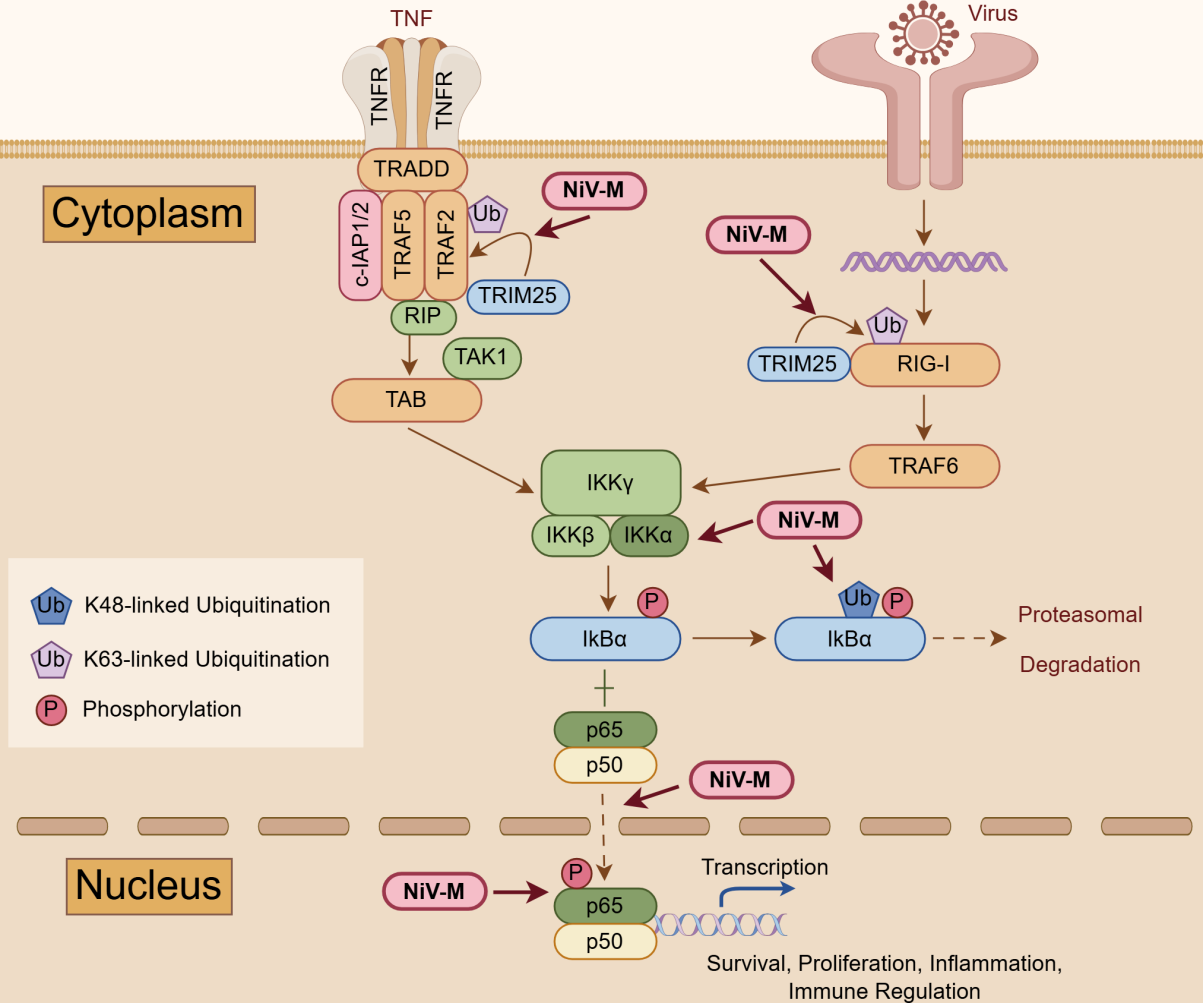
Schematic representation of the mechanisms by which NiV-M hijacks the classical NF-κB pathway. NiV-M interacts with multiple cellular signaling molecules in the pathway, including TRIM25, IKKα, IκBα, and p65. Specifically, NiV-M enhances the E3 ubiquitin ligase activity of TRIM25, facilitating its interaction with both RIG-I and TRAF2 and promoting their K63-linked ubiquitination, thereby amplifying upstream signaling. Furthermore, NiV-M strengthens the interaction between IKKα and IKKβ, enhancing IKK complex formation and activity, which leads to increased phosphorylation of IκBα. NiV-M also promotes K48-linked polyubiquitination of IκBα, targeting it for proteasomal degradation. The degradation of IκBα releases the p50/p65 NF-κB dimer, enabling its nuclear translocation. NiV-M further promotes p65 phosphorylation, amplifying NF-κB-driven gene expression. Collectively, these interactions enable NiV-M to potently and synergistically activate NF-κB-driven gene expression, which may contribute to the severe immunopathology and high lethality associated with NiV infection.

The NiV-M, a highly pathogenic *paramyxovirus*, is a multifunctional structural protein traditionally recognized for its essential role in viral assembly and budding by mediating the association of viral structural components with the host cell membrane (44, 47, 48). However, emerging evidence, including the data presented in this study, significantly expands its functional repertoire, demonstrating its direct involvement in modulating host innate immune signaling pathways, including inhibition of the type I interferon response mediated by IKKε (18). Our findings elucidated that NiV-M is not merely a structural component but also a potent virulence factor that strategically manipulates the NF-κB signaling cascade, a central regulator of inflammation and antiviral responses (Fig. 6). We demonstrated that NiV-M achieves this through a coordinated strategy, targeting multiple critical nodes within the NF-κB cascade, from upstream signaling complexes to the nuclear translocation of the p65. The finding that NiV-M interacts directly with several core components of the pathway, namely TRIM25, IKKα, IκBα, and p65, suggests that it acts as a molecular scaffold or enhancer to facilitate signal propagation. Notably, NiV-M overexpression significantly suppressed the replication of influenza A virus (PR8), a finding suggesting that NiV-M-activated NF-κB signaling can induce an antiviral state in specific cellular contexts. In addition, we observed that the P, C, N, F, and G proteins also significantly enhanced TNFα-induced activation of the NF-κB pathway (Fig. 1A). Although the enhancement by each was modest, the cumulative effect of these proteins may represent one of the underlying mechanisms that underpin the high pathogenicity of NiV. This finding thus suggests that the virus deploys a cooperative strategy to orchestrate the systematic modulation of the host inflammatory microenvironment.

A key contribution of this study is the discovery that NiV-M promotes NF-κB activation by hijacking the E3 ubiquitin ligase TRIM25 and potentially interacting with the BBOX/CCD domain of TRIM25, which mediates protein interactions and multimerization (41). By strengthening the interaction between TRIM25 and both RIG-I and TRAF2, NiV-M promotes the K63-linked ubiquitination of these essential cellular signaling molecules. Ubiquitination serves as a critical regulatory switch: for RIG-I, ubiquitination initiates antiviral signaling (39) and regulates virus-induced NF-κB activation, whereas ubiquitination of TRAF2 enhances its interaction with TAK1 or IKKβ to regulate TNFα-induced NF-κB activation (40). These results underscore the biological importance of NiV-M-mediated regulation of the NF-κB pathway through TRIM25.

Activated IKK complexes phosphorylate IκBα at serine residues 32 and 36, promoting its K48-linked polyubiquitination and subsequent proteasomal degradation (46). Phosphorylation of p65 (e.g., at Ser536) and its nuclear translocation are hallmarks of NF-κB activation. Upon TNFα stimulation, the IKKα/β complex phosphorylates IκBα, leading to K48-linked ubiquitination and subsequent degradation of IκBα, thereby releasing p65 and enabling its nuclear entry and transcriptional activity. Another major contribution was the finding that NiV-M interacts with IKKα and promotes its association with IKKβ, thereby facilitating assembly and activation of the core IKK complex. This function of NiV-M is crucial for NF-κB activation and is demonstrated in two aspects: first, NiV-M facilitates the formation of a stable and highly active IKK complex, providing the structural foundation for downstream signaling. Second, the enhanced dimerization of IKKα and IKKβ promotes efficient trans-phosphorylation of their activation loops, a critical step for efficient kinase activity. This results in rapid and robust phosphorylation and degradation of IκBα, thereby enabling nuclear translocation of NF-κB. Consequently, by co-opting these cellular signaling molecules, NiV-M efficiently activates the pro-survival and inflammatory functions of NF-κB, which may support viral replication and immune evasion. In addition, NiV-M promotes p65 phosphorylation and nuclear translocation. Collectively, these findings demonstrate that NiV-M regulates the NF-κB pathway at multiple nodes, functioning not only to activate NF-κB but also to govern NF-κB-mediated transcription.

Fatal cases caused by NiV infection are strongly associated with excessive inflammatory responses, often characterized by severe systemic vasculitis and encephalitis (6, 7). The transcription factor NF-κB is a central regulator of pro-inflammatory signaling and is implicated in driving the pathological inflammation observed in severe viral infections (49). However, the mechanistic relationship between NiV infection and the NF-κB pathway remains poorly defined. A significant knowledge gap exists regarding how specific NiV proteins modulate NF-κB to contribute to viral pathogenesis and immune evasion. Our study addresses this gap by investigating the interplay between NiV and NF-κB signaling. The regulation of the canonical NF-κB pathway by NiV-M suggests a deliberate strategy to exploit pro-survival and inflammatory signaling. Our findings indicate that NiV-M enhances hyperactivation of the NF-κB pathway, a process that may underlie the severe immunopathology and high virulence of NiV infection. Hyperactivation of NF-κB likely provides a favorable environment for viral replication by suppressing apoptosis and simultaneously disrupting the precise temporal regulation of inflammatory genes, thereby contributing to the immunopathology and cytokine dysregulation observed in NiV infection. Endothelial damage, vasculitis, endothelial inflammation, microinfarctions, and encephalitis (1, 6, 50, 51), documented in autopsy studies, may be exacerbated by NF-κB-driven inflammatory mediators.

Our findings provide evidence for the multifaceted role of NiV-M in regulating the NF-κB pathway, while several limitations of this study should be acknowledged. First, most experiments were conducted in HEK293T cells, which, although offering high transfection efficiency and reproducibility, may not fully recapitulate the physiology of primary human endothelial cells or neurons, the primary targets of NiV infection, where pathogenesis of encephalitis and respiratory disease manifests (6, 50, 52). Therefore, the physiological relevance of our findings needs to be further validated in more disease-relevant cell culture systems and, ultimately, in *in vivo* models. Second, our study focuses primarily on the mechanistic role of NiV-M in initiating NF-κB signaling. However, the downstream consequences of this hyperactivation during infection remain insufficiently characterized, and the global host transcriptome should be further investigated. Finally, while our study identifies critical nodes such as IKK and TRIM25, the therapeutic potential of targeting these virus–host interactions was not explored.

In conclusion, our data support a model in which NiV-M is the principal regulator of NF-κB signaling among all NiV-encoded proteins. NiV-M interacts with multiple cellular signaling molecules within the NF-κB pathway. Specifically, NiV-M promotes TRIM25-mediated ubiquitination of RIG-I and TRAF2, enhances IKK complex activity, accelerates IκBα degradation, and promotes p65 phosphorylation and nuclear translocation, thereby amplifying NF-κB-driven gene expression (Fig. 6). These findings uncover a multifaceted strategy employed by NiV-M to modulate the NF-κB pathway and further highlight its role in driving NiV pathogenesis via dysregulation of host inflammatory responses. Moreover, they suggest potential therapeutic approaches for alleviating NiV-induced immunopathology by targeting virus–host interactions.

## MATERIALS AND METHODS

### Reagents, antibodies, and plasmids

Recombinant Human TNFα Protein (catalog no. RP00993) was purchased from ABclonal Biotechnology (Wuhan, China).

Mouse anti-Myc-Tag mAb (AE010), rabbit anti-V5-Tag mAb (AE089), NF-κB p65/RelA Rabbit mAb (A19653), IKKα Rabbit pAb (A2062), IκBα Rabbit mAb (A19714), phospho-NF-κB p65/RelA-S536 Rabbit mAb (AP1294), phospho-IκBα-S32 Rabbit mAb (AP0707), HRP-conjugated Goat anti-Mouse IgG (H + L) (AS003), HRP-conjugated Goat anti-Rabbit IgG (H + L) (AS014), and HRP-conjugated Goat anti-Mouse IgG Light Chain (AS062) were purchased from ABclonal Biotechnology (Wuhan, China). HA tag Polyclonal antibody (51064-2-AP), GAPDH Monoclonal antibody (60004-1-Ig), and HRP-conjugated IgG Fraction Monoclonal Mouse Anti-Rabbit IgG, Light Chain Specific (SA00001-7L) were purchased from Proteintech (Wuhan, China). DYKDDDDK (Flag) Tag Mouse mAb (RA1003-01) was purchased from Vazyme (Nanjing, China). Lamin A/C (4C11) Mouse mAb (4777S) was purchased from Cell Signaling Technology (Danvers, MA, USA). Anti-TRIM25/EFP Rabbit mAb (ab167154) was purchased from Abcam (Cambridge, MA, USA). Normal mouse IgG (sc-2025) was purchased from Santa Cruz Biotechnology (Santa Cruz, CA, USA).

Plasmids expressing NiV viral protein genes, named pCA-P, pCA-V, pCA-W, pCA-C, pCA-L, pCA-M, pCA-N, pCA-F, and pCA-G, respectively, were provided by Professor Wuxiang Guan from the Wuhan Institute of Virology (WIV), Chinese Academy of Science (CAS). Expression plasmids for V5-NiV-M, Flag-NiV-M, HA-NiV-M, and GFP-NiV-M were amplified by PCR from pCA-M and cloned into the expression vectors pCAGGS-V5, pCAGGS-Flag, pCAGGS-HA, and pEGFP-N1 using the ClonExpress II One Step Cloning Kit (Vazyme, Wuhan, China). Flag-NSUN2 (P66288), Myc-RIP1 (P64531), Flag-TRIM25 (P3543), Flag-TAK1 (P37967), HA-TAB1 (P24624), Flag-IKKα (P54856), HA-IκBα (P50403), GFP-Flag-IκBα (P66437), Flag-p50 (P56017), HA-p65 (P40120), and EGFP-N1(P0133) expression plasmids were purchased from MiaoLing Plasmid Platform (Wuhan, China). Myc-TRIM25 was amplified by PCR amplification from Flag-TRIM25 and cloned into the expression vectors pCAGGS-Myc using the ClonExpress II One Step Cloning Kit (Vazyme, Wuhan, China). Reporter plasmids, including NF-κB-Luc (carrying the NF-κB response element upstream of the firefly luciferase (Luc) reporter gene), pRL-TK (expressing Renilla Luc), HA-TRAF6, HA-RIG-I, RIG-IN-Flag, Flag-TRAF2, HA-Ub-WT, HA-Ub-K48, HA-Ub-K63, HA-IKKα, Flag-IKKβ, and HA-IKKγ, were kindly provided by Wang Hanzhong (WIV, CAS). Myc-HeV-M and Flag-RAD18 were kindly provided by Professor Chen Mingzhou from Hubei University. Different TRIM25 truncated derivatives were cloned from Flag-TRIM25 using the ClonExpress II One Step Cloning Kit (Vazyme, Wuhan, China). All plasmids were verified by DNA sequencing at Sangon Biotech (Shanghai, China).

### Cells, virus, and transfection

Human embryonic kidney 293T (HEK293T, ATCC CRL-3216) cells, human lung epithelial cells (A549), and HeLa cells were cultured in Dulbecco’s modified Eagle medium (Gibco, USA) with 10% fetal bovine serum at 37°C in a 5% CO_2_ humidified incubator. The IAV employed in this study was A/Puerto Rico/8/1934 (PR8/H1N1), a strain maintained in our laboratory. Transfections were performed using Lipofectamine 8000 (C0533FT; Beyotime Biotechnology, Shanghai, China) or Lipofectamine 2000 (Invitrogen, Carlsbad, CA, USA) according to the manufacturer’s instructions. siRNA targeting TRIM25 was transfected using Lipofectamine 2000.

### Dual-luciferase reporter assay

HEK293T cells were seeded in 24-well plates at a density of 6 × 10^4^ cells per well. After overnight incubation, cells were co-transfected with NF-κB luciferase reporter plasmid (120 ng per well) and the internal control Renilla luciferase (30 ng per well), together with 250–500 ng of empty vector as a control or plasmids encoding Flag-NiV-M or V5-NiV-M, and the indicated adaptor genes. At 24 h post-transfection (hpt), cells were harvested in passive lysis buffer (Promega, Madison, WI, USA). The cell lysates were evaluated using the Dual-Luciferase Assay Kit (Promega, Madison, WI, USA) according to the manufacturer’s protocol. All experiments were performed in triplicate and repeated at least three times.

### Confocal microscopy

HeLa cells were seeded in glass-bottom plates and transfected with plasmids. At 24 hpt, cells were fixed with 4% paraformaldehyde (PFA) for 20 min, permeabilized with 0.5% Triton X-100 in PBS for 15 min, and blocked with 5% bovine serum albumin (BSA) for 1 h. After blocking, cells were incubated with primary antibody for 2 h at room temperature, followed by three washes with PBST and incubation with appropriate Alexa Fluor-conjugated secondary antibody for 1 h. Following three additional washes, nuclei were stained with 4′,6-diamidino-2-phenylindole (DAPI) for 15 min. Images were acquired using a confocal microscope.

### Coimmunoprecipitation assay

HEK293T cells were seeded in 6-well plates at a density of 5 × 10^5^ cells per well and transfected with the indicated plasmids using Lipofectamine 8000 according to the manufacturer’s instructions. At 24 hpt, cells were washed with cold phosphate-buffered saline (PBS) and lysed in lysis buffer (P0013; Beyotime Biotechnology, Shanghai, China) containing protease inhibitor cocktail (HY-K0010; MedChemExpress, Shanghai, China) and PMSF (HY-B0496; MedChemExpress, Shanghai, China) on ice for 10 min. For immunoprecipitation (IP) experiments, the supernatant was then incubated directly with anti-Flag magnetic beads (HY-K0207; MedChemExpress, Shanghai, China) or anti-HA magnetic beads (HY-K0201; MedChemExpress, Shanghai, China) at 4°C overnight with gentle rotation. Alternatively, before cell lysis, Myc, V5, or IgG antibodies were incubated with Protein A/G magnetic beads (HY-K0202; MedChemExpress, Shanghai, China) in PBS at 4°C for at least 4 h. The antibody-conjugated beads were washed thrice with PBST (HY-K1022; MedChemExpress, Shanghai, China). Subsequently, the supernatant was mixed with the antibody-bound beads, followed by incubation overnight at 4°C with gentle rotation. Beads were subsequently washed five times with PBST, resuspended in 1 × SDS loading buffer, boiled for 10 min, and proteins were analyzed by Western blotting.

### Generation of TRIM25-knockout HeLa cell lines

TRIM25-knockout (TRIM25-KO) HeLa cell lines were generated using the CRISPR-Cas9 system. The single-guide RNA (sgRNA) sequence targeting the TRIM25 gene (5′-CACCAAGCACGTCTTCACGG-3′) was cloned into the LentiCRISPRv2-GFP vector. For lentivirus production, the viruses were generated as described before (53). HeLa cells were seeded in six-well plates and transduced with the harvested lentivirus. At 24 h post-infection (hpi), selection was initiated with 3 µg/mL puromycin. Positive monoclonal cell lines were isolated by fluorescence-activated cell sorting (FACS) based on GFP fluorescence intensity. Knockout efficiency was confirmed at the protein level by Western blot analysis.

### *In vitro* kinase assay

HEK293T cells were transfected with plasmids encoding HA-IκBα, Flag-IKKα, or V5-NiV-M, individually. At 36 hpt, cells were harvested, and the respective proteins were purified by immunoprecipitation using anti-HA, anti-Flag, or anti-V5 antibody-conjugated beads. Equal amounts of the substrate HA-IκBα were then incubated with or without Flag-IKKα or V5-NiV-M in kinase buffer (9802, Cell Signaling Technology, Danvers, MA, USA) in the presence or absence of ATP (9804, Cell Signaling Technology, Danvers, MA, USA) at 30°C for 1 h. Reactions were terminated by adding 5 × SDS loading buffer (CW0027S, CWBIO, Taizhou, China) to a final concentration of 1×, followed by boiling for 10 min. The proteins were subsequently analyzed by Western blotting.

### Nuclear and cytoplasmic fractionation

Cytoplasmic and nuclear proteins from HEK293T cells were extracted according to the manufacturer’s instructions (P0027; Beyotime Biotechnology, Shanghai, China). Briefly, HEK293T cells were seeded in six-well plates. After 14 h, the cells were transfected with either a plasmid expressing NiV-M or an empty vector. At 24 hpt, the cells were stimulated with TNFα for the indicated time. Subsequently, cytoplasmic and nuclear proteins were isolated using the commercial fractionation kit according to the manufacturer’s instructions.

### RNA isolation and RT-PCR

Total RNA was extracted using the Total RNA Extraction Kit (Magen Biotechnology, China). The expression of each gene was measured with Hiscript III RT SuperMix for qPCR (+gDNA wiper) (R323, Vazyme) and ChamQ Universal SYBR qPCR Master Mix (Vazyme), according to our previous study (54). The primer sequences for IL-6, IL-8, and TNFα were designed according to protocols outlined in a previous study (40).

### Statistical analysis

Cells were enumerated to categorize distinct p65 subcellular localization profiles (nuclear, cytoplasmic, and nucleocytoplasmic). The proportion of cells displaying each pattern was normalized and quantified as a percentage of the total counted cell population. Immunoblot band densitometry was analyzed with ImageJ software. Values represent the mean ± standard deviation (SD) of triplicate experiments. Statistical comparisons were performed with an unpaired Student’s t-test in GraphPad Prism 6, where asterisks denote significance levels (*P < 0.05, **P < 0.01, ***P < 0.001, ****P < 0.0001).

## Data Availability

The data that support the findings of this study are available from the corresponding authors upon request.

## SUPPLEMENTAL MATERIAL

Fig. S1 to S3 jvi.00185-26-s0001.docx qPCR and co-IP results.

ASM does not own the copyrights to Supplemental Material that may be linked to, or accessed through, an article. The authors have granted ASM a non-exclusive, world-wide license to publish the Supplemental Material files. Please contact the corresponding author directly for reuse.
