# Supplementary material for: Nipah virus matrix protein promotes NF-κB activation by targeting multiple signaling modulators
Source: J Virol. 2026 May 21;100(6):e00185-26. doi: 10.1128/jvi.00185-26 (PMC13288614; doi:10.1128/jvi.00185-26)
Supplement: Fig. S1 to S3 — qPCR and co-IP results. [file jvi.00185-26-s0001.docx]

**Nipah virus matrix protein promotes NF-κB activation**

**by targeting multiple signaling modulators**

**Supplemental Files**


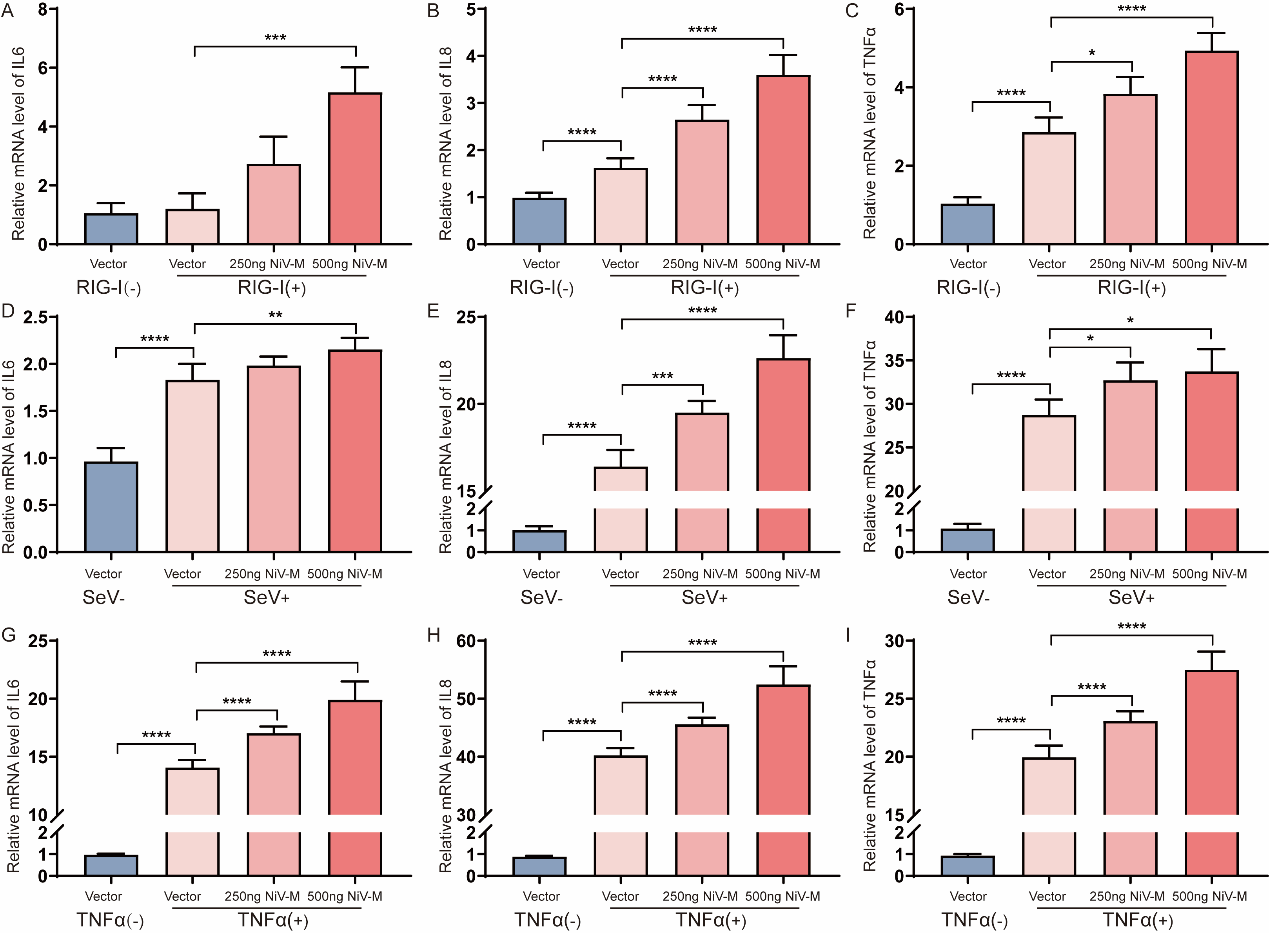


Fig S1. Effect of NiV-M on the expression of proinflammatory cytokines in HEK293T cells.

(A-C) HEK293T cells were co-transfected with RIG-I, empty vector or increasing amounts of NiV-M plasmid as indicated. At 24 hpt, cells were lysed for RNA extraction, and qPCR was performed to detect the expression of IL-6 (A), IL-8 (B), and TNFα (C). (D-F) HEK293T cells were transfected with empty vector or increasing amounts of NiV-M plasmid as indicated. At 24 hpt, cells were infected with SeV (100 HA unit/mL) for 16 h, mRNA levels of IL-6 (D), IL-8 (E) and TNFα (F) were measured by qPCR analysis. (G-I) HEK293T cells were transfected as in panels D-F. At 24 hpt, cells were treated with TNFα (10 ng/mL) for 8 h, followed by RNA extraction and qPCR analysis to measure IL-6 (G), IL-8 (H), and TNFα (I) expression. **P* < 0.05, ***P* < 0.01, ****P* < 0.001, *****P* < 0.0001.


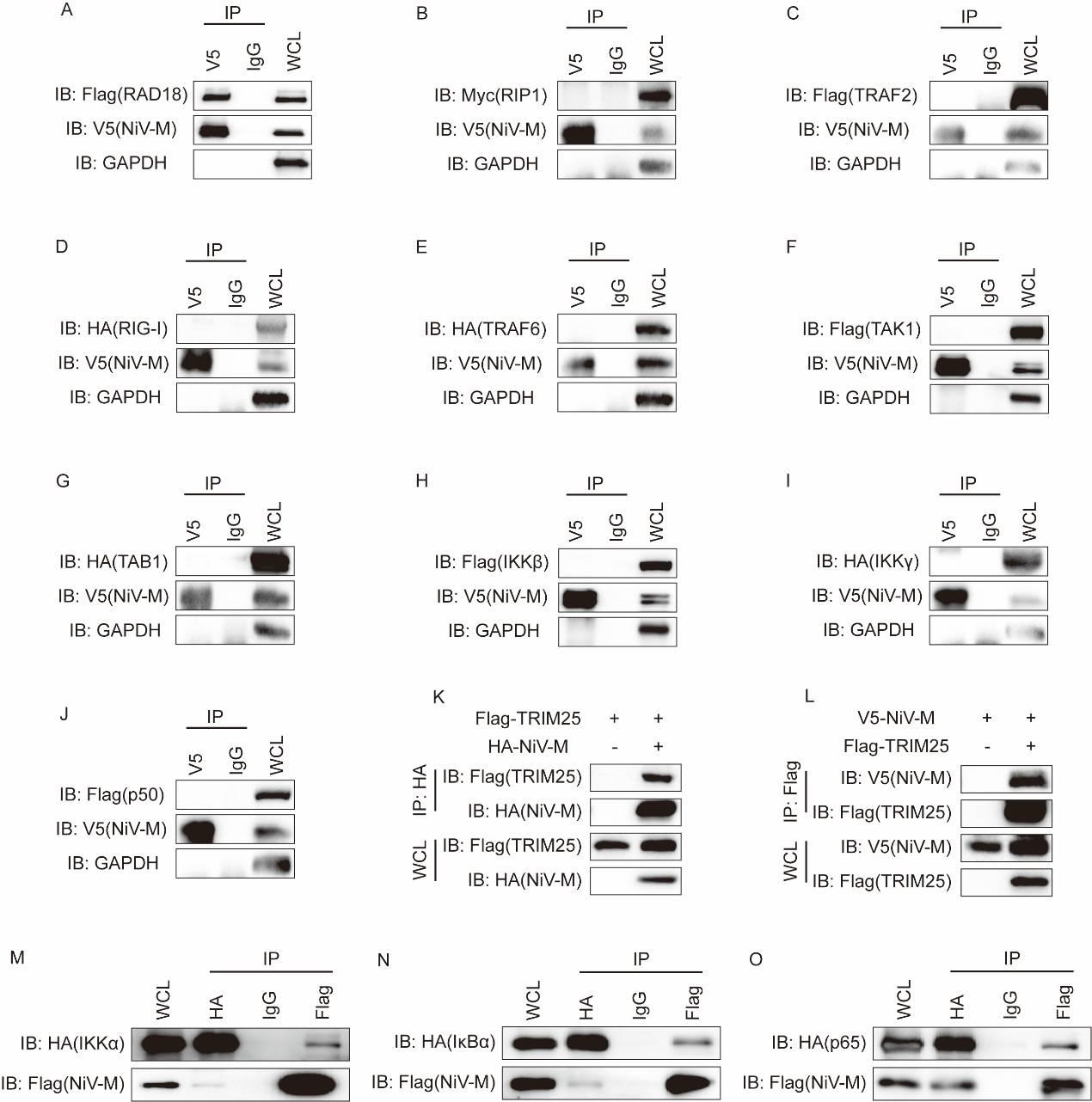


Fig S2. Interaction analysis between NiV-M and RAD18, RIP1, TRAF2, RIG-I, TRAF6, TAK1, TAB1, IKKβ, IKKγ, p50, TRIM25, IKKα, IκBα, or p65.

HEK293T cells were co-transfected with V5-tagged NiV-M and expression plasmids for RAD18 (A) RIP1 (B), TRAF2 (C), RIG-I (D), TRAF6 (E), TAK1 (F), TAB1 (G), IKKβ (H), IKKγ (I), or p50 (J). At 24 hpt, cells were collected and subjected to immunoprecipitation using anti-V5 antibody-conjugated beads (IP: V5) or non-specific IgG (IP: IgG) as a control. Cells were co-transfected with Flag-TRIM25 and HA-NiV-M or V5-NiV-M. At 24 hpt, lysates were immunoprecipitated using anti-HA (IP: HA) (K) or anti-Flag (IP: Flag) (L) antibody-conjugated beads. HEK293T cells were co-transfected with Flag-NiV-M and HA-tagged IKKα (M), IκBα (N) or p65 (O). At 24 hpt, lysates were immunoprecipitated using anti-HA (IP: HA), IgG (IP: IgG), or anti-Flag (IP: Flag) antibody-conjugated beads. Western blotting analysis was carried out with the indicated antibodies to evaluate IP complexes and WCL.


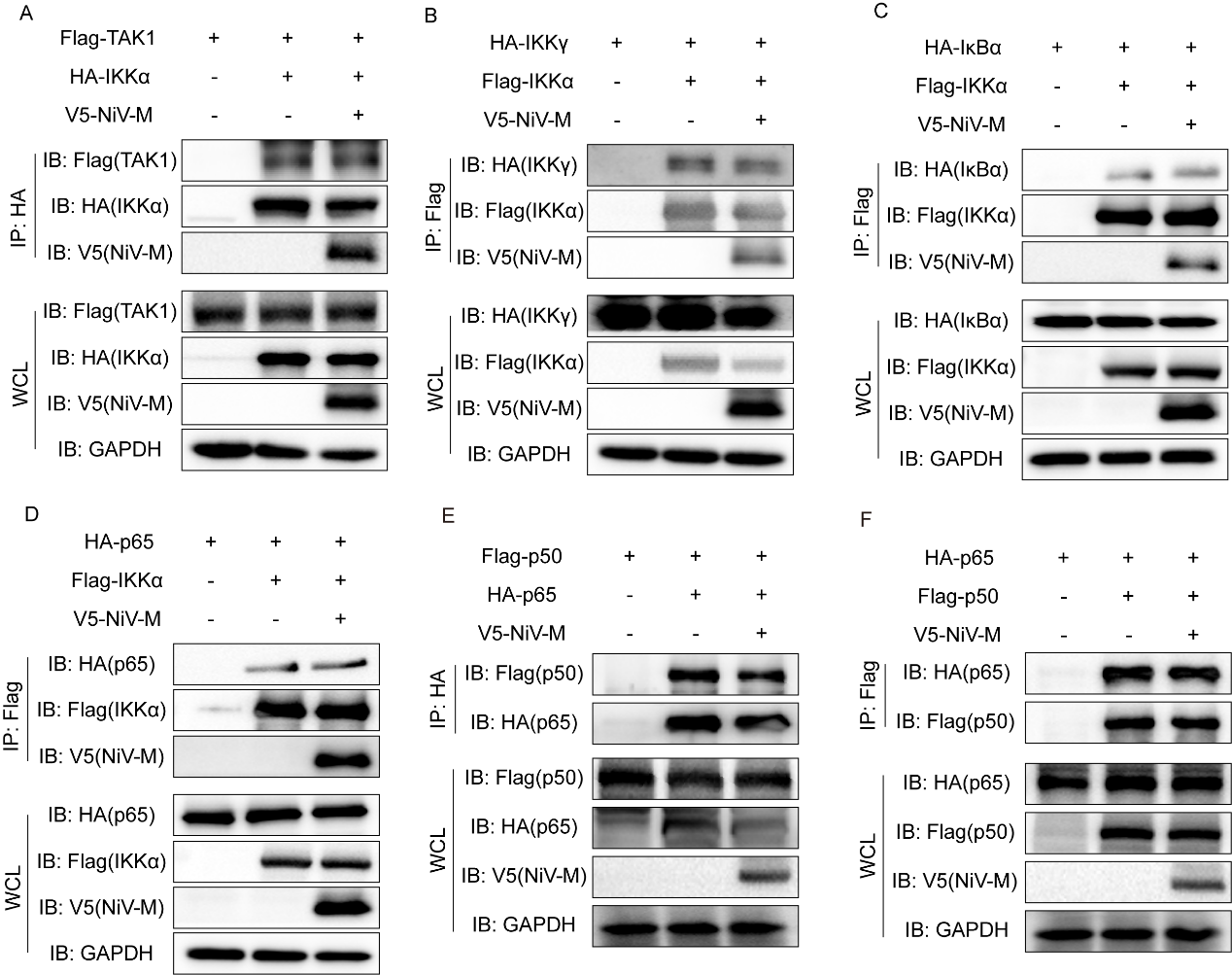


Fig S3. The effects of NiV-M on interactions between signaling modulators.

HEK293T cells were transfected with different combinations of plasmids encoding HA- or Flag-tagged TAK1, IKKγ, IκBα or p65, together with Flag- or HA-tagged IKKα and either V5-NiV-M or empty vector. Cell lysates were harvested at 24 hpt and subjected to immunoprecipitation using anti-HA antibody-conjugated beads (IP: HA) (A) or anti-Flag antibody-conjugated beads (IP: Flag) (B-D). HEK293T cells were transfected with plasmids encoding HA-p65, Flag-p50, and either V5-NiV-M or empty vector. Cell lysates were harvested at 24 hpt and subjected to immunoprecipitation using anti-HA antibody-conjugated beads (IP: HA) (E) or anti-Flag antibody-conjugated beads (IP: Flag) (F).
